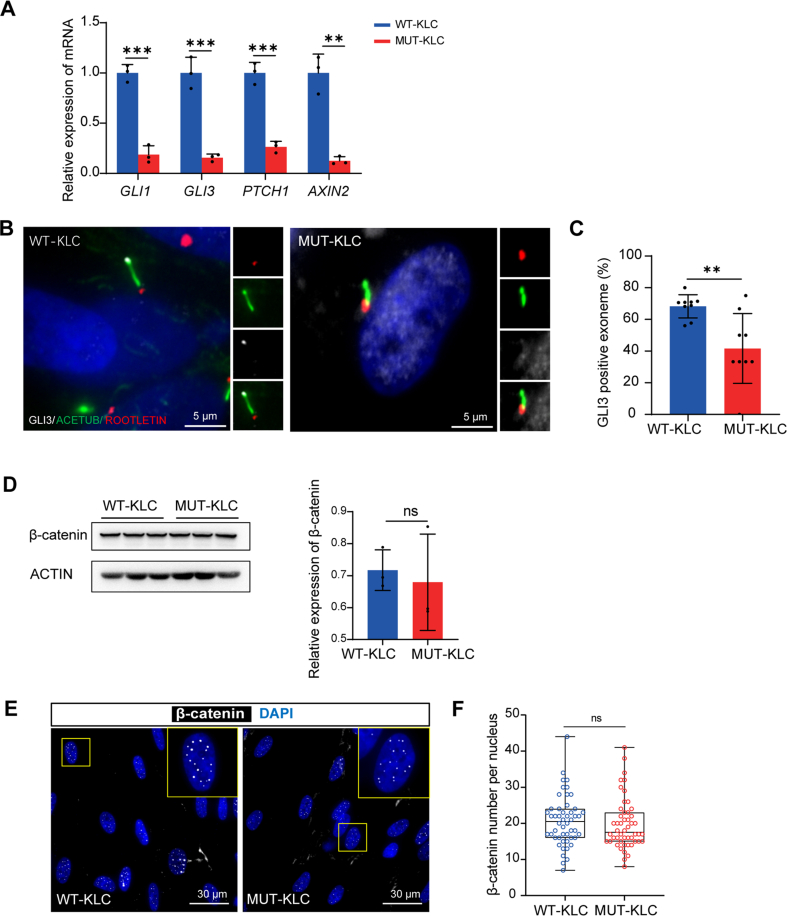# Compound heterozygous mutations in *BBS7* cause kidney abnormalities in Bardet-Biedl syndrome

**DOI:** 10.1016/j.gendis.2025.101792

**Published:** 2025-08-07

**Authors:** Jie Min, Rong Xiao, Qian Fu, Yue Huang, Hui Wang

**Affiliations:** a Department of Nephrology, Key Laboratory of Basic and Clinical Pediatric Nephrology, Baoding Hospital of Beijing Children's Hospital, Capital Medical University, Regional Center for Children's Health, Baoding, Hebei 071030, China; b Department of Nephrology, Key Laboratory of Major Diseases in Children, Ministry of Education, Beijing Children's Hospital, Capital Medical University, National Center for Children's Health, Beijing 100045, China; c State Key Laboratory of Common Mechanism Research for Major Diseases, Institute of Basic Medical Sciences, Chinese Academy of Medical Sciences and Peking Union Medical College, Beijing 100005, China; d Department of Medical Genetics, Institute of Basic Medical Sciences, Chinese Academy of Medical Sciences and Peking Union Medical College, Beijing 100005, China

Bardet-Biedl syndrome (BBS) is a rare autosomal recessive ciliopathy characterized predominantly by renal abnormalities, which constitute the leading cause of early mortality in patients with BBS. Genotype–phenotype analyses indicate that mutations in genes encoding components of the BBSome core complex (BBS2-BBS7-BBS9) correlate with higher penetrance of renal manifestations^1^; however, the underlying pathogenic mechanisms remain unclear. Within the core complex, the BBS7 protein occupies a unique and pivotal role, directly interacting with chaperone complexes and initiating BBSome assembly.^2^ Studies utilizing mouse models carrying *BBS7* mutations have failed to fully recapitulate the human BBS phenotype, suggesting either species-specific differences or distinct pathogenic thresholds in ciliopathies.^3^ Thus, establishing genetically consistent, humanized disease models is essential. Human induced pluripotent stem cells (hiPSCs) derived from patients carry disease-specific genetic and epigenetic information, rendering them ideal tools for modeling various disorders, including BBS. In this study, we identify a novel *BBS7* mutation, c.754G > A(p.D252N), and established a kidney lineage model from patient-specific hiPSCs harboring compound heterozygous mutations in the *BBS7* gene, aimed at investigating the effects of *BBS7* mutations on renal development and elucidating the underlying pathogenic mechanisms.

The patient enrolled in this study presented with polyuria, polydipsia (>3 years), and foamy urine (1 year). Renal function assessment revealed chronic kidney disease (CKD) stage 3 (eGFR: 33.02 mL/min/1.73 m^2^). Ultrasound revealed bilateral renal hypoplasia and dysplasia, characterized by increased parenchymal echogenicity, indistinct corticomedullary differentiation, localized calyceal abnormalities, and a small cyst at the left kidney's upper pole.Additional clinical features are summarized (Table S1), confirming BBS diagnosis.

Genetic analysis identified compound heterozygous BBS7 mutations: a novel maternally inherited missense variant c.754G > A(p.D252N) and a known pathogenic paternally inherited splice donor mutation c.849+1G > C (Fig. 1A). SpliceAI strongly predicted (99%) that c.849+1G > C disrupts normal mRNA splicing (Fig. S1A). The novel c.754G > A variant was classified as a variant of uncertain significance (VUS) by ACMG guidelines, requiring functional validation.

**Figure 1:**
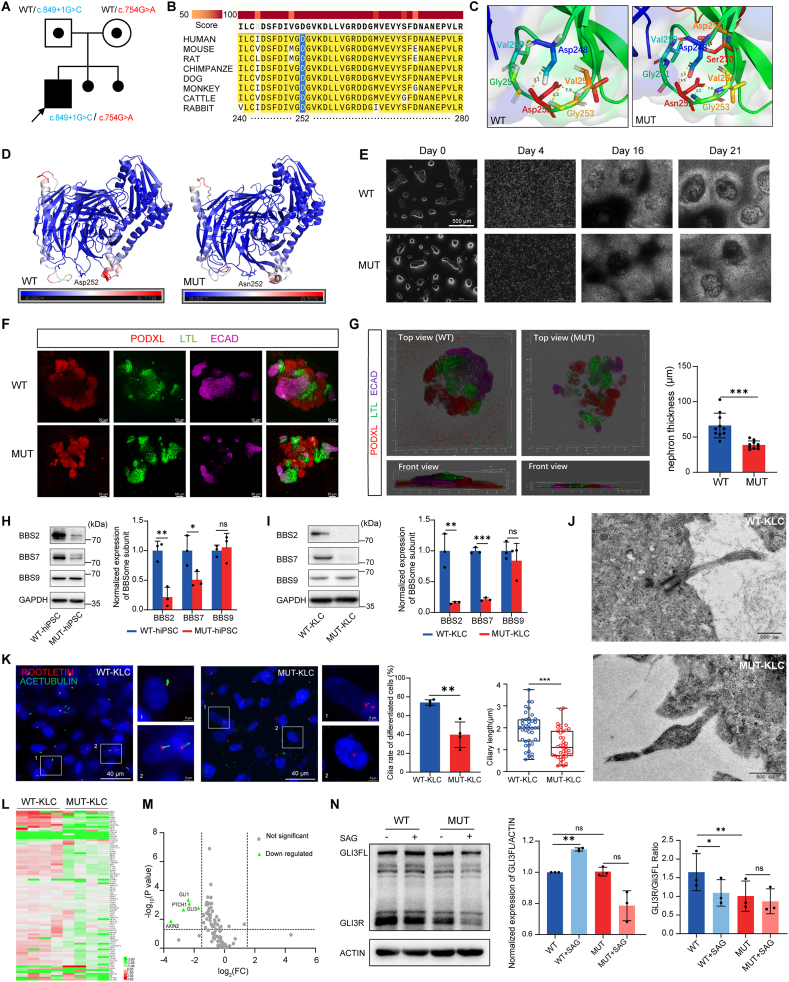
Identification and functional characterization of *BBS7* mutations associated with renal abnormalities. **(A)** Pedigree diagram illustrating the segregation of *BBS7* mutations within the proband's family. The black arrow denotes the proband. Black circles represent two fetuses aborted due to renal cystic changes detected during prenatal examinations. **(B)** Sequence alignment demonstrating the conservation of Asp252 (highlighted in blue) and flanking residues across eukaryotic BBS7 orthologs. **(C)** Local structure and hydrogen-bond network around the D252N mutation site of the BBS7 protein. Yellow dashed lines indicate hydrogen bonds, with numbers denoting bond lengths (in angstroms, Å). **(D)** Flexibility clustering analysis of WT and MUT BBS7 proteins. Protein structures are depicted in a cartoon model, with color gradients indicating residue flexibility (blue denotes regions of low flexibility and high stability; red denotes regions of high flexibility and greater fluctuation). **(E)** Representative bright-field images showing differentiation of KLCs at indicated time points; scale bar, 500 μm **(F)** Immunocytochemical staining of nephron-like structures for markers of podocytes (PODXL), proximal tubules (LTL), and distal tubules (CDH1) on day 28; scale bar, 50 μm **(G)** Representative front and top views, along with thickness quantification, of three-dimensional reconstructed nephron-like structures. **(H–I)** Western blot analysis of BBS2, BBS7, and BBS9 protein expression levels in hiPSCs (H) and KLCs (I). **(J)** Transmission electron microscopy (TEM) images showing longitudinal sections of primary cilia in KLCs. **(K)** Representative immunofluorescence images and quantitative analyses of primary cilia in WT and MUT KLCs. ACETUBULIN (acetylated α-tubulin) marks the ciliary axoneme, and rootletin marks the ciliary base. **(L)** Heatmap of RT^2^ Profiler PCR Array analysis comparing primary cilia gene expression between WT and MUT KLCs. **(M)** Volcano plot of differential ciliary gene expression; gray dots denote genes with no significant difference between WT and MUT groups, while green dots highlight genes significantly downregulated in the MUT group. **(N)** Western blot analysis showing GLI3 protein expression in WT and MUT KLCs, treated with or without 500 nM SAG for 8 h.

The strong evolutionary conservation of residue D252 and its adjacent residues among eukaryotic BBS7 orthologs suggests their potential functional importance (Fig. 1B). Molecular dynamics simulations indicate that, although the D252N variant did not affect the overall conformation or stability of the BBS7 protein significantly (Fig. S1B–D), it altered the local hydrogen-bond interaction network between the mutation site and neighboring residues, and reduced the flexibility of secondary structures surrounding this region (Fig. 1C and D). Consequently, these local structure changes may impair essential conformational transitions necessary for the biological functions of the protein.

We previously generated patient-derived hiPSCs (MUT-hiPSCs) from peripheral blood mononuclear cells.^4^ To investigate *BBS7* mutation effects on renal development, MUT-hiPSCs and control-derived wild-type hiPSCs (WT-hiPSCs) were differentiated into kidney lineage cells using a 2D culture system (Fig. S2A).

We observed that both groups of cells spontaneously formed nephron-like structures during differentiation (Fig. 1E), and key nephron-segment markers—including the podocyte marker PODXL, proximal tubule marker LTL, water channel marker AQP1, distal tubule marker ECAD, and collecting duct marker GATA3—maintained typical sequential expression patterns (Fig. 1F; Fig. S2B). However, nephron-like structures derived from MUT-hiPSCs exhibited significant abnormalities, characterized by decreased structural thickness and reduced compactness compared to WT controls (Fig. 1G). Such defects in spatial self-organization, including abnormal cellular arrangement and loss of polarity, are consistent to some degree with renal structural abnormalities (renal hypoplastic dysplasia) observed clinically in the patient via ultrasonography. Additionally, following primary culture, mutant kidney lineage cells (MUT-KLCs) exhibited significantly reduced proliferative and migratory capacities compared to wild-type kidney lineage cells (WT-KLCs) at the same passage (Fig. S3A–D). This reduction in cellular viability may contribute to their diminished capacity for self-organization.

Further analysis of the impact of *BBS7* mutations on the BBSome core complex revealed markedly reduced BBS7 protein expression in MUT-hiPSCs even prior to differentiation, with further pronounced decreases observed post-differentiation. Among the other two core subunits of the BBSome complex examined, BBS2 exhibited significantly reduced protein expression in MUT cells, whereas BBS9 protein levels remained relatively stable throughout differentiation (Fig. 1H and I).

To explore the mechanism underlying the simultaneous degradation of BBS2 and BBS7 proteins, molecular docking, and molecular dynamics simulations were performed to assess the impact of the D252N variant on their interaction. Our results indicated that BBS7 and BBS2 can form stable complexes (Table S2); however, the D252N mutation altered the interaction interface between the two proteins (Fig. S4A). Specifically, molecular docking analyses predicted that in the wild-type complex, the binding sites of BBS2 and the E3 ubiquitin ligase membrane associated Ring–CH–type finger 1 (MARCHF1) on BBS7 spatially overlapped. In contrast, following the D252N mutation, the predicted binding site for MARCHF1 shifted to a separate, non-overlapping region distinct from the BBS2 binding interface (Fig. S4B). This spatial rearrangement may increase the accessibility of the BBS7-BBS2 complex to MARCHF1, thereby potentially facilitating ubiquitin-mediated degradation. These findings provide a plausible explanation for the simultaneous degradation of BBS2 and BBS7 proteins resulting from the *BBS7* mutation.

Considering that BBS is a ciliopathy, we examined the ultrastructure of KLCs by transmission electron microscopy, revealing characteristic bulging in the mid-region of primary cilia in MUT-KLCs compared to WT-KLCs (Fig. 1J). Immunofluorescence analysis of intraflagellar transport protein IFT88 showed abnormal accumulation along ciliary shafts in MUT-KLCs, particularly near the tips, suggesting partially impaired intraflagellar trafficking (Fig. S5A and B). Further immunostaining demonstrated significantly decreased ciliogenesis and shortened cilia in MUT-derived cells, indicating defective ciliary assembly or maintenance due to *BBS7* mutations (Fig. 1K).

Primary cilia function as crucial hubs for cellular signal transduction, and structural or functional abnormalities can disrupt multiple signaling pathways. To investigate the impact of *BBS7* mutations on ciliary function, we performed a primary cilia-focused RT2 Profiler PCR Array analysis. The analysis revealed extensive downregulation of genes involved in primary cilia functions and associated signaling pathways (Fig. 1L). Specifically, the expression of *GLI1*, *GLI3*, and *PTCH1*—key components of the SHH pathway—as well as *AXIN2*, a critical regulator of the canonical WNT pathway, was significantly reduced in MUT-KLCs (Fig. 1M; Fig. S6A).

GLI3 is a crucial downstream transcription factor in the SHH pathway, and exists in either an activated full-length form (GLI3FL) or a repressor form (GLI3R), both of which are tightly regulated by primary cilia.^5^ Our analysis demonstrated a reduced number of GLI3-positive cilia in MUT-KLCs (Fig. S6B and C). Functional assays following treatment with the SHH pathway agonist SAG revealed markedly diminished responsiveness in MUT cells, as indicated by the unchanged GLI3R/GLI3FL ratio (Fig. 1N), further highlighting dysfunctional SHH signaling in *BBS7*-mutated cells.

β-catenin (encoded by *CTNNB1*) is crucial for canonical WNT signaling, and its nuclear translocation is promoted by cilia absence.^5^ However, we observed that primary cilia abnormalities caused by *BBS7* mutations did not affect β-catenin nuclear localization or stability (Fig. S6D–F). Whether other components or aspects of WNT signaling are affected by *BBS7* mutations remains to be explored. Detailed antibody information and primer sequences can be found in Table S3 and Table S4, respectively.

The limitations of our study include the reliance on a cellular model derived from a single patient and the absence of mutation-rescue experiments using gene editing. Nonetheless, we identified and characterized the pathogenicity of a novel *BBS7* mutation (c.754G > A,p.D252N), thereby expanding the known mutational spectrum of this gene. Importantly, our findings provide enhanced mechanistic insight into ciliopathies and establish a valuable human-derived cellular model for future studies, potentially informing precision medicine strategies for renal manifestations of BBS.

## CRediT authorship contribution statement

**Jie Min:** Writing – review & editing, Writing – original draft, Validation, Software, Resources, Methodology, Investigation, Data curation, Conceptualization. **Rong Xiao:** Writing – original draft, Software, Methodology, Investigation, Data curation. **Qian Fu:** Writing – original draft, Visualization, Methodology, Investigation, Data curation. **Yue Huang:** Writing – review & editing, Visualization, Validation, Supervision, Resources, Methodology, Investigation, Formal analysis. **Hui Wang:** Writing – review & editing, Visualization, Validation, Supervision, Resources, Project administration, Methodology, Investigation, Funding acquisition, Conceptualization.

## Ethics approval and consent to participate

This study received approval from the Ethical Committee of Baoding Hospital of Beijing Children's Hospital [2022-15], Capital Medical University, and was conducted in accordance with the ethical guidelines outlined in the Declaration of Helsinki. Written informed consent was obtained from all participants.

## Funding

This work was supported by the 10.13039/501100003787Hebei Natural Science Foundation (China) (H2022104021).

## Conflict of interests

The authors have declared that no conflict of interest exists.

## Figures and Tables

**figs1:**
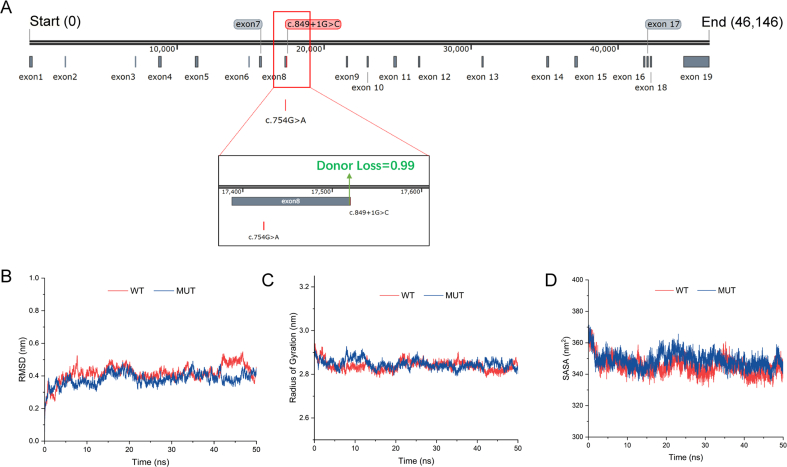


**figs2:**
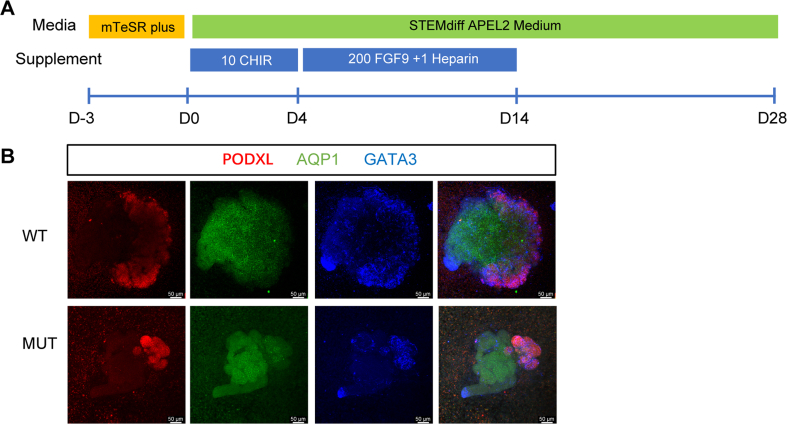


**figs3:**
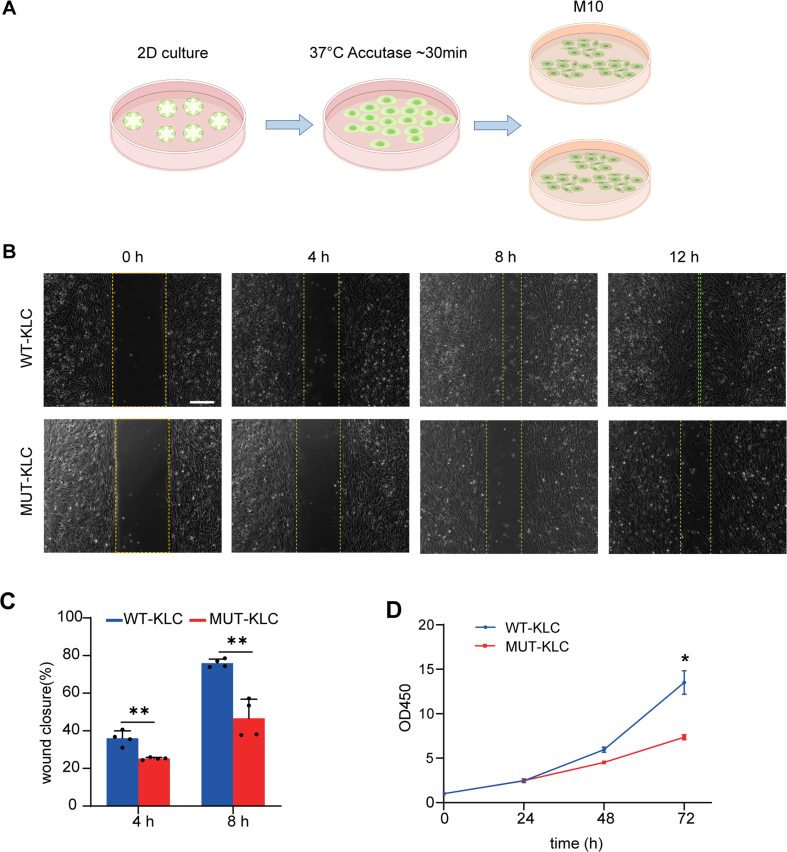


**figs4:**
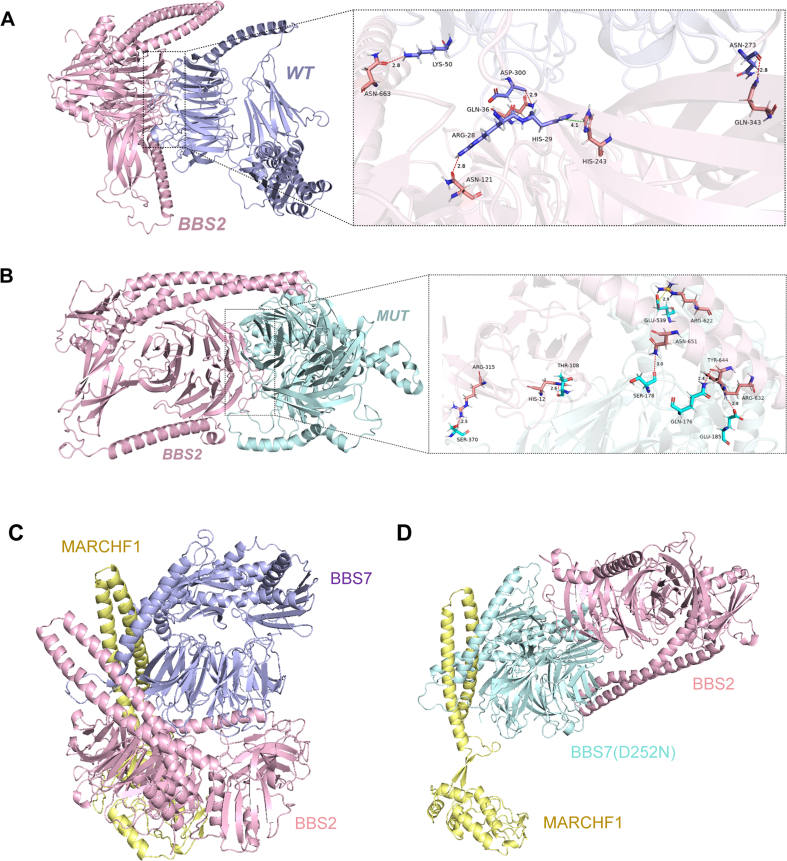


**figs5:**
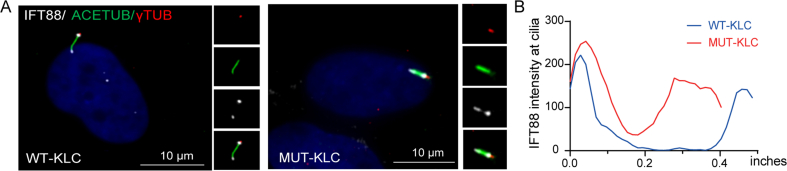


**figs6:**