# Supplementary material for: Compound heterozygous mutations in BBS7 cause kidney abnormalities in Bardet-Biedl syndrome
Source: Genes Dis. 2025 Aug 7;13(3):101792. doi: 10.1016/j.gendis.2025.101792 (PMC12874413; doi:10.1016/j.gendis.2025.101792)
Supplement: Multimedia component 1 [file mmc1.docx]

**Materials and Methods**

***Clinical Data Collection and Genetic Analysis***

General clinical data and renal characteristics were obtained from electronic medical records, including demographic details, chief complaint, present illness history, growth and developmental milestones, physical examination findings, laboratory test results, and imaging findings. Diagnosis of BBS was independently confirmed by two experienced pediatric nephrologists based on established diagnostic criteria (Table S1) ^[1]^. Variant pathogenicity was assessed according to the guidelines of the American College of Medical Genetics and Genomics (ACMG) ^[2]^.

***Sequence Alignment***

Amino acid sequences of BBS7 homologs were retrieved from the NCBI database, including Homo sapiens (NP_789794.1), Mus musculus (NP_082086.2), Rattus norvegicus (NP_001012180.1), Pan troglodytes (XP_024212045.1), Canis lupus familiaris (XP_533301.2), Macaca mulatta (XP_001101234.2), Bos taurus (NP_001178275.2), and Oryctolagus cuniculus (XP_008266137.2). Multiple-sequence alignment was performed using SnapGene software.

***Protein Structure Modeling***

The structure of wild-type BBS7 protein was retrieved from UniProt (accession: Q8IWZ6). The mutant BBS7 (p.D252N) structure was generated by homology modeling using SWISS-MODEL and evaluated using the Qualitative Model Energy Analysis (QMEAN) scoring function, yielding a high-quality model (QMEAN = 0.85). The impact of the D252N mutation on hydrogen bond interactions was analyzed using PyMOL software (Version 2.5.0a0, Schrödinger, LLC).

***Molecular Dynamics Simulation***

All-atom molecular dynamics (MD) simulations of wild-type (WT) and mutant (MUT) proteins were performed using GROMACS (v2023.3 for system preparation; v2020.6 for simulations). Protein structures were parameterized using the AMBER force field and solvated in a cubic TIP3P water box extending 10 Å from the protein surface. Na^+^ and Cl^−^ ions were added to achieve physiological ionic strength (~0.145 mol/L NaCl). Following energy minimization using the steepest-descent method (force tolerance 1000 kJ/mol·nm), systems were equilibrated for 100 ps each under NVT (310.15 K) and NPT (1 bar, Berendsen barostat) conditions. Production simulations were run for 50 ns with a timestep of 2 fs. Hydrogen bond lengths were constrained with the LINCS algorithm, electrostatic interactions were calculated using Particle-Mesh Ewald (PME, cutoff 1.2 nm), and non-bonded pair lists were updated every 10 steps with a 10 Å cutoff. Post-simulation analyses included RMSD, RMSF, radius of gyration (Rg), hydrogen bonds, and solvent-accessible surface area (SASA). All software tools used (PyMOL, GROMACS, AlphaFold, etc.) are freely available under open-source licenses.

***Molecular Docking***

Rigid-body docking between BBS7 and BBS2 proteins was initially performed using HDOCK to identify favorable orientations. Subsequently, flexible docking refinement was conducted using RosettaDock, and optimal conformations were selected based on Rosetta’s scoring function. Protein-protein interaction interfaces and contacts were analyzed with the Protein–Ligand Interaction Profiler (PLIP), and docking conformations were visualized using PyMOL.

***Binding Free Energy Calculation***

The binding free energies between proteins were calculated using the Molecular Mechanics/Generalized Born Surface Area (MM/GBSA) method. The binding free energy (ΔG_bind_) was computed according to the following equation:

ΔG_bind_ = G_complex_−(G_receptor_+G_ligand_) = ΔE_vdW_+ΔE_elec_+ΔG_GB_+ΔG_SA_​

ΔE_vdW_ and ΔE_elec_ respectively represent van der Waals interaction and electrostatic interaction. ΔG_GB_ and ΔG_SA_ collectively represent solvation free energies, where ΔG_GB_ corresponds to polar solvation free energy calculated using the GB model developed by Nguyen et al. (*igb* = 2) ^[3]^. The nonpolar solvation energy, ΔG_SA_, was calculated from the solvent accessible surface area (SASA) and surface tension (γ), according to the following relationship:

ΔG_SA_ = 0.0072 × ΔSASA^[4]^

***Origin and Culture Methods of hiPSCs***

The methodology for generating the hiPSC line from a male patient affected by BBS has been described previously^[5]^. Previous research indicates that hiPSCs derived from different tissue sources (fibroblasts and peripheral blood mononuclear cells) exhibit similar transcriptomic, epigenomic, and differentiation characteristics^[6]^. The WT-hiPSC line was obtained from the Cell Bank/Stem Cell Bank of the Chinese Academy of Sciences (catalog number: SCSP-1301, cell line name: DYR0100), reprogrammed from fibroblasts derived from the foreskin tissue of a healthy male neonate. hiPSCs were cultured in 24-well or 6-well plates coated with Matrigel® matrix (BD Biosciences, #356231) using mTeSR™ Plus medium (StemCell Technologies, #100-0274), with medium changes every two days. Cells were passaged by treating with Accutase solution (Sigma-Aldrich, #A6964), and reseeded in mTeSR™ Plus medium supplemented with 10 μM ROCK inhibitor Y-27632 (Selleck Chemicals, #S1049) for the first 24 hours.

***Kidney Lineage Cells Differentiation***

Differentiation of kidney lineage cells (KLCs) in 2D culture was performed following protocols described previously by Bantounas et al. (2018) and Morizane et al. (2015) (Fig. S2A) [7,8]. hiPSCs were seeded onto Matrigel® matrix-coated plates at a density of 1,500–2,000 cells/cm² in mTeSR™ Plus medium supplemented with 10 μM ROCK inhibitor Y-27632. After 24 hours, cultures were maintained for an additional two days without Y-27632. Starting from day 4, the medium was changed to STEMdiff™ APEL™2 Medium (StemCell Technologies, #05275) supplemented with 10 μM CHIR99021 (Selleck Chemicals, #S2924) for four days, followed by STEMdiff™ APEL™2 Medium containing 200 ng/mL FGF9 (Sino Biological, #10262-HNAE) and 1 μg/mL heparin (Sigma-Aldrich, #3149) for another ten days. Subsequently, cultures were maintained in basal STEMdiff™ APEL™2 Medium without additional growth factors for an additional 14 days to facilitate primary KLC formation.

***Culture of Kidney Lineage Cells***

Differentiated KLCs were maintained as primary cells in Dulbecco's Modified Eagle Medium (DMEM, Gibco, #11995073) supplemented with 10% fetal bovine serum (FBS, Vivacell, #C04001), GlutaMAX™ Supplement (Gibco, #35050061), minimum essential medium non-essential amino acids (Gibco, #11140050), 2-mercaptoethanol (Gibco, #21985023), and penicillin-streptomycin (Gibco, #15140122). Cells were dissociated using Accutase solution (Sigma-Aldrich, #A6964) and subcultured at a ratio of 1:2 to generate first-passage (P1) cells (Fig. S3A). Due to limitations in primary cell expansion, all experiments involving differentiated cells from both groups were conducted at matched passages, not exceeding five passages.

***Prediction of E3 Ubiquitin Ligases and Protein Interaction Analysis***

Potential E3 ubiquitin ligases targeting BBS7 were predicted using UbiBrowser 2.0, and membrane-associated RING-CH-type finger 1 (MARCHF1) was selected based on the high confidence scores. The structure of MARCHF1 was predicted using AlphaFold3 and optimized via energy minimization with the Rosetta Relax module. Protein–protein docking between BBS7 and MARCHF1 was initially performed using HDOCK, followed by refinement with RosettaDock. Optimal complexes were selected based on docking scores and the rationality of the interface. PyMOL (Version 2.5.0a0, Schrödinger, LLC) was employed to structurally align wild-type and mutant (D252N) BBS7 complexes with MARCHF1 and BBS2 to evaluate conformational changes and exposure of potential ubiquitination sites.

***Immunocytochemistry***

Cells were fixed with 4% paraformaldehyde (Servicebio, #G1101) for 15 minutes at room temperature and washed three times with phosphate-buffered saline (PBS). Subsequently, cells were permeabilized with 0.3% Triton X-100 for 10 minutes, blocked with 5% goat serum (ZSGB-BIO, #ZLI-9021) or donkey serum (Abbkine, #BMS0140) in 0.1% Triton X-100/PBS for 1 hour at room temperature, and incubated with primary antibodies overnight at 4°C. Fluorophore-conjugated secondary antibodies were applied and incubated for 1 hour at room temperature. For staining with biotinylated Lotus tetragonolobus lectin (LTL; Vector Laboratories, #B-1325), endogenous biotin receptors and streptavidin binding sites were blocked using a Streptavidin/Biotin Blocking Kit (Vector Laboratories) prior to incubation with LTL. Details of primary antibodies and their dilution ratios are provided in Table S3. Images were captured and reconstructed three-dimensionally using a Leica confocal microscope. All immunofluorescence analyses were independently repeated at least three times with similar results, and representative images were presented.

***Transmission Electron Microscopy (TEM)***

KLCs were fixed overnight at 4°C in 2.5% glutaraldehyde, followed by three washes (15 min each) with 0.1 M phosphate buffer (pH 7.0). Samples were post-fixed with 1% osmium tetroxide solution for 1–2 h and washed again three times (15 min each) in 0.1 M phosphate buffer (pH 7.0). Samples were dehydrated through a graded ethanol series (30%, 50%, 70%, 80%, 90%, and 100%), with each step lasting 15 min, and transitioned into pure acetone for 20 min. Subsequently, samples underwent infiltration using graded mixtures of acetone and resin at ratios of 3:1 for 2 h, 1:1 for 3 h, 1:3 for 3 h, and finally pure resin overnight. Resin embedding was polymerized by gradually heating from 35°C to 80°C. Ultrathin sections (~70–90 nm) were prepared using a Leica EM UC7 ultramicrotome and stained sequentially with uranyl acetate for 15 min and lead citrate (pH ~12) for 5 min. Sections were then dried and imaged using a transmission electron microscope (FEI Tecnai G2 12).

***Reverse Transcription PCR and Quantitative Real-Time PCR***

Total RNA was extracted using TRIzol™ Reagent (Thermo Fisher Scientific, #15596018). Approximately 1 μg of total RNA was reverse-transcribed into cDNA using HiScript III RT SuperMix (Vazyme, #R323-01). Conventional PCR was conducted post-reverse transcription to verify primer specificity, and PCR products were analyzed by electrophoresis on a 1.5% agarose gel. Quantitative real-time PCR (RT-qPCR) was performed using SYBR™ Green PCR Master Mix (Thermo Fisher Scientific, #A57156) on a CFX Connect Real-Time PCR System (Bio-Rad), according to the manufacturer's protocol. Primers used for RT-qPCR analysis are listed in Table S4. Relative expression levels were normalized to the housekeeping gene GAPDH (Sangon Biotech, #B661104).

***Western Blot***

Cells harvested by digestion with Accutase were washed twice with phosphate-buffered saline (PBS) and lysed on ice for 1 h using RIPA lysis buffer (Beyotime, #P0013). Lysates were centrifuged at 12,000×g for 15 min at 4 °C, and supernatants were collected. Protein concentrations were determined using a Pierce BCA Protein Assay Kit (Thermo Fisher Scientific, #23225), and samples were adjusted to equal concentrations. Protein samples (20 μg per lane) were mixed with loading buffer, denatured at 95°C for 10 min, separated by SDS-PAGE, and transferred onto a 0.2 μm nitrocellulose membrane (Bio-Rad, #1620097) via wet transfer. Membranes were blocked with 5% skim milk for 1 h at room temperature, followed by incubation with primary antibodies diluted in antibody dilution buffer overnight at 4°C. Membranes were washed three times with Tris-buffered saline with 0.1% Tween-20 (TBST) and incubated with HRP-conjugated secondary antibodies against mouse, rabbit, or goat IgG for 1 h at room temperature. After three additional washes with TBST, proteins were visualized using SuperSignal™ West Pico PLUS Chemiluminescent Substrate (Thermo Fisher Scientific, #34580).

***Primary Cilia Immunohistochemical Imaging and Quantification***

KLCs were serum-starved for 24 hours in DMEM (Gibco, #11966025) without serum or other supplements prior to cilia staining. The cilia staining procedure was identical to the cell immunofluorescence protocol described previously, with antibody details listed in Table S3. Primary cilia were imaged using a Leica DMi8 wide-field inverted microscope equipped with Leica LAS X software. Cilia length and fluorescence intensity of ciliary proteins were quantified using Leica LAS X software (3.7.4.23463) and ImageJ software (NIH), respectively. Cilia length was defined as the distance from the basal body to the axoneme tip. All samples were processed uniformly during staining and imaging. Quantitative analyses were performed based on data obtained from at least three experiments.

***Cell Counting Kit-8 (CCK8) assays***

Cell proliferation was assessed using the Cell Counting Kit-8 (CCK-8; NCM Biotech, #C6050). Briefly, KLCs from matched passages of WT and MUT groups were seeded into 96-well plates at a density of 6,000 cells per well. Cells were cultured at 37°C with 5% CO₂, and proliferation was evaluated at 24 h, 48 h, and 72 h post-seeding. At each time point, cells were incubated with 10 μL of CCK-8 reagent per well for 3 h, and absorbance was measured at 450 nm using a microplate reader. All experiments were performed independently in triplicate.

***Cell Migration (Scratch Wound) Assay***

Scratch wound migration assays were performed using two-well culture inserts (Ibidi, Germany). Briefly, KLC suspensions from matched passages of WT and MUT groups were seeded at 5 × 10^4^ cells per compartment (100 μL at 5 × 10^5^ cells/mL) and incubated at 37°C for 24 hours to form confluent monolayers. The inserts were then carefully removed with sterilized forceps to generate a uniform 500 μm wound gap. Floating cells were removed by washing twice with Dulbecco’s phosphate-buffered saline (DPBS), followed by adding 1 mL DMEM supplemented with 1% FBS into each dish. Images were captured immediately after scratch creation (0 h) using a Zeiss inverted microscope (10× objective), and positions were marked for subsequent imaging. Cells were cultured further, and images were captured at the marked positions at 4, 8, and 12 hours post-scratch. The wound area was quantified using ImageJ software, and the percentage of wound closure was calculated as follows:

wound closure (%) = [(original scratch area - final scratch area) /original scratch area] × 100

All experiments were performed independently at least three times.

***Statistical Analysis***

All statistical analyses were performed using GraphPad Prism version 8.0 (GraphPad Software). Data are primarily presented as means ± standard deviations (SD), with selected results depicted using box plots showing medians and quartiles. Data were analyzed assuming a normal distribution. Unpaired two-tailed t-tests were used for comparisons between independent samples, while paired two-tailed t-tests were employed for matched samples. Statistical significance was defined as *P* < 0.05, with significance levels denoted as follows: *P* < 0.05 (*), *P* < 0.01 (**), *P* < 0.001 (***).

***Reference***

[1] Dollfus H, Lilien MR, Maffei P, et al. Bardet-Biedl syndrome improved diagnosis criteria and management: Inter European Reference Networks consensus statement and recommendations. Eur J Hum Genet. 2024;32(11):1347-1360.

[2] Richards S, Aziz N, Bale S, et al. Standards and guidelines for the interpretation of sequence variants: a joint consensus recommendation of the American College of Medical Genetics and Genomics and the Association for Molecular Pathology. Genet Med. 2015;17(5):405-424.

[3] Nguyen H, Roe DR, Simmerling C. Improved Generalized Born Solvent Model Parameters for Protein Simulations. J Chem Theory Comput. 2013;9(4):2020-2034.

[4] Weiser J, Shenkin P S, Still W C. Approximate atomic surfaces from linear combinations of pairwise overlaps (LCPO)[J]. J Comput Chem, 1999, 20(2): 217-230.

[5] Fu Q, Wang H, Zhou N, et al. Generation of a human iPSC line from a Bardet-Biedl syndrome patient compound heterozygous for the BBS7 variants c.849 + 1G > C/c.754G > A. Stem Cell Res. 2021;54:102428.

[6] Kyttälä A, Moraghebi R, Valensisi C, et al. Genetic Variability Overrides the Impact of Parental Cell Type and Determines iPSC Differentiation Potential. Stem Cell Reports. 2016;6(2):200-212.

[7] Bantounas I, Ranjzad P, Tengku F, et al. Generation of Functioning Nephrons by Implanting Human Pluripotent Stem Cell-Derived Kidney Progenitors. Stem Cell Reports. 2018;10(3):766-779.

[8] Morizane R, Lam AQ, Freedman BS, Kishi S, Valerius MT, Bonventre JV. Nephron organoids derived from human pluripotent stem cells model kidney development and injury. Nat Biotechnol. 2015;33(11):1193-1200.
