# Supplementary material for: Compound heterozygous mutations in BBS7 cause kidney abnormalities in Bardet-Biedl syndrome
Source: Genes Dis. 2025 Aug 7;13(3):101792. doi: 10.1016/j.gendis.2025.101792 (PMC12874413; doi:10.1016/j.gendis.2025.101792)
Supplement: Multimedia component 2 [file mmc2.docx]

| **Table S1 Clinical characteristics of the patient diagnosed with BBS in this study** | |
| --- | --- |
| Characteristic | Information |
| Gender | Boy |
| Age at the first evaluation | 12 years |
| Primary diagnostic features of BBS |  |
| Retinal degeneration | + |
| Polydactyly | + |
| Obesity | - |
| Learning disorders/cognitive impairment | + |
| Hypogonadism/genital abnormalities | + |
| Renal disease | + |
| Secondary diagnostic features of BBS |  |
| Language barriers | + |
| Strabismus/astigmatism/cataract | + |
| Brachydactyly/syndactyly | - |
| Developmental delay | + |
| Polyuria/polydipsia (nephrogenic diabetes insipidus) | + |
| Ataxia/Spasticity | - |
| Diabetes mellitus | - |
| Dental anomalies | - |
| Congenital heart disease | - |
| Liver and other gastrointestinal abnormalities | - |
