# Supplementary material for: Compound heterozygous mutations in BBS7 cause kidney abnormalities in Bardet-Biedl syndrome
Source: Genes Dis. 2025 Aug 7;13(3):101792. doi: 10.1016/j.gendis.2025.101792 (PMC12874413; doi:10.1016/j.gendis.2025.101792)
Supplement: Multimedia component 3 [file mmc3.docx]

**Table S2 Binding free energy analysis of wild-type and mutant BBS2-BBS7 complex**

| **Energy（kcal/mol）** | **WT** | **MUT** |
| --- | --- | --- |
| Δ*E*_vdW_ | -247.50 | -185.97 |
| Δ*E*_ele_ | 111.88 | 123.54 |
| Δ*E*_GB_ | -63.22 | -84.40 |
| Δ*E*_surf_ | -34.13 | -24.60 |
| Δ*E*_Gas_ | -135.63 | -62.43 |
| Δ*E*_solv_ | -97.35 | -109.00 |
| Δ*E*_Bind_ | -232.97 | -171.43 |

Δ*E*_vdW_, van der Waals interaction energy; Δ*E*_ele_, electrostatic interaction energy; Δ*E*_GB_, polar solvation free energy; Δ*E*_surf_, nonpolar solvation free energy; Δ*E*_Gas_, gas-phase interaction energy, Δ*E*_Gas_ = Δ*E*_vdW_ + Δ*E*_ele_; Δ*E*_solv_, solvation free energy, Δ*E*_solv_ = Δ*E*_GB_ + Δ*E*_surf_; Δ*E*_Bind_total binding free energy, Δ*E*_Bind_ = Δ*E*_Gas_ + Δ*E*_solv_.
