# Supplementary material for: Compound heterozygous mutations in BBS7 cause kidney abnormalities in Bardet-Biedl syndrome
Source: Genes Dis. 2025 Aug 7;13(3):101792. doi: 10.1016/j.gendis.2025.101792 (PMC12874413; doi:10.1016/j.gendis.2025.101792)
Supplement: Multimedia component 4 [file mmc4.docx]

| **Table S3. List of antibodies** | | | | |
| --- | --- | --- | --- | --- |
| Antibody | Species | Dilution | Source | Catalog No. |
| PODXL | Mouse | 1:200 | Thermofisher | 39-3800 |
| LTL | Biotinylated | 1:200 | Vector laboratories | B-1325-2 |
| ECAD | Rabbit | 1:200 | Cell signaling | 3195 |
| AQP1 | Rabbit | 1:200 | Sigma-Aldrich | SAB5200109 |
| GATA3 | Goat | 1:50 | R&D Systems | AF2605 |
| Acetylated-α-tubulin | Mouse | 1:200 | Proteintech | 66200-1-Ig |
| γ-tubulin | Mouse | 1:200 | Proteintech | 66320-1-Ig |
| Rootletin | Rabbit | 1:200 | Abcam | ab121653 |
| IFT88 | Rabbit | IF 1:200 | Proteintech | 13967-1-AP |
| BBS2 | Rabbit | 1:1000 | Proteintech | 11188-2-AP |
| BBS7 | Rabbit | 1:1000 | Proteintech | 13458-1-AP |
| BBS9 | Rabbit | 1:1000 | Proteintech | 14460-1-AP |
| GLI3 | Goat | IF 1:50  WB 1:1000 | R&D Systems | AF3690 |
| β-catenin | Rabbit | IF 1:200  WB 1:5000 | Proteintech | 51067-2-AP |
| Goat lgG Antibody | - | 1:1000 | R&D Systems | HAF019 |
| Goat anti-mlgG1-AF488 | - | 1:1000 | Invitrogen | A-21121 |
| Goat anti-mlgG2a-AF555 | - | 1:1000 | Invitrogen | A-21137 |
