# Supplementary material for: Compound heterozygous mutations in BBS7 cause kidney abnormalities in Bardet-Biedl syndrome
Source: Genes Dis. 2025 Aug 7;13(3):101792. doi: 10.1016/j.gendis.2025.101792 (PMC12874413; doi:10.1016/j.gendis.2025.101792)
Supplement: Multimedia component 5 [file mmc5.docx]

| **Table S4. RT-PCR and qPCR primers.** | | |
| --- | --- | --- |
| Gene | Forward Primer | Reverse Primer |
| *OCT4* | TTCAGCCAAACGACCATCTG | TCAGCTTCCTCCACCCACTT |
| *NANOG* | CCCCAGCCTTTACTCTTCCTA | CCAGGTTGAATTGTTCCAGGTC |
| *GLI1* | CCCAACTCCACAGGCATACAGG | ACAGATTCAGGCTCACGCTTCTC |
| *GLI3* | GAAGTGCTCCACTCGAACAGA | GTGGCTGCATAGTGATTGCG |
| *PTCH1* | ACCATCCTCGGCGTTCTCAATG | CGTTGGCTGGAGACACCTCAG |
| *AXIN2* | GGTTTCCCCTTGGACCTCG | CCGTCGAAGTCTCACCTTTAATG |
