# Supplementary material for: Compound heterozygous mutations in BBS7 cause kidney abnormalities in Bardet-Biedl syndrome
Source: Genes Dis. 2025 Aug 7;13(3):101792. doi: 10.1016/j.gendis.2025.101792 (PMC12874413; doi:10.1016/j.gendis.2025.101792)
Supplement: Multimedia component 6 [file mmc6.pdf]

保定市儿童医院临床科研项目伦理审批件

2022（年）伦审【科】第（15）号

研究项目名称	BBS7 基因在 iPSC 分化的肾小管细胞内的致病性及功能研究				
项目负责人	王辉	开展项目科室	肾脏内科	职称	主任医师
项目审查类别	☑ 科研立项 ☐ 多中心合作项目 ☐ 专利申请及成果转化<br>☐ 其他（请注明）：				
药物/器械/体外诊断试剂名称	NA				
审查方式	☑ 会议审查 ☐ 加快审查 ☐ 免除审查				
审阅及批准文　件	1、项目资料清单<br>2、伦理审查申请表<br>3、临床研究方案（版本号：1.0 版，版本日期：2022 年 2 月 27 日）<br>4、知情同意书（版本号：1.0 版，版本日期：2022 年 2 月 27 日）<br>5、主要研究者组成及履历				
	本医学伦理委员会以《纽伦堡法典》、《赫尔辛基宣言》、《涉及人的生物医学研究伦理审查办法》、医学国际组织理事会及世界卫生组织的有关文献为指导原则，并受中国有关法律法规、制度政策及道德规范的约束。<br>经本伦理委员会审查，同意开展研究。<br>研究涉及采集、保存、利用、对外提供我国人类遗传资源的情况，需向人类遗传资源管理办公室进行申报，获得批准后方可开展研究。<br>研究过程中若变更主要研究者，对临床研究方案、知情同意书、招募材料等的任何修改，请研究者提交修正方案伦理审查申请表，获得批准后执行。				

<table>
<tr><td>医学伦理委员会审查意见</td><td colspan="3">发生严重不良事件，请研究者及时提交严重不良事件报告。
请按照医学伦理委员会规定的年度/定期跟踪审查率，研究者在截止日期前提交本中心研究进展报告。
研究纳入了不符合纳入标准或符合排除标准的受试者，符合终止试验规定而未让受试者退出研究，给予错误治疗或剂量，给予方案禁止的合并用药等没有遵从方案开展研究的情况，请研究者提交违背方案报告。
研究暂停/提前终止，请研究者及时提交暂停/终止研究报告。
完成研究后，请研究者提交结题报告。

主任委员签名：
签名日期：2022.3.4
（盖章）保定市儿童医院 医学伦理委员会</td></tr>
<tr><td>年度/定期跟踪审查频率</td><td>12 个月</td><td>截止日期</td><td>2023 年 3 月 4 日</td></tr>
<tr><td>批件有效期</td><td>1 年</td><td>截止日期</td><td>2023 年 3 月 4 日</td></tr>
<tr><td>审查结果</td><td colspan="3">（√）同意　（　）作必要修正后同意　（　）作必要修正后重审
（　）不同意　（　）终止或暂停已批准的试验　（　）弃权</td></tr>
</table>
