# Supplementary material for: Compound heterozygous mutations in BBS7 cause kidney abnormalities in Bardet-Biedl syndrome
Source: Genes Dis. 2025 Aug 7;13(3):101792. doi: 10.1016/j.gendis.2025.101792 (PMC12874413; doi:10.1016/j.gendis.2025.101792)
Supplement: Multimedia component 7 [file mmc7.docx]

**Supplementary Figure Legends**

**Figure S1. Genomic schematic of BBS7 mutations and molecular dynamics simulations**

A. Genomic schematic of the BBS7 gene. The patient's two mutation sites (c.849+1G>C and c.754G>A) are indicated by red vertical lines, with the c.849+1G>C splice donor site mutation highlighted by a green arrow. The BBS7 gene spans 46,146 nucleotides and contains 19 exons (exons 1–19), represented by gray boxes.

B. Root Mean Square Deviation (RMSD) curves for wild-type (WT, blue) and mutant D252N (MUT, red) BBS7 proteins during 50 ns molecular dynamics simulations. RMSD values (y-axis) are plotted against simulation time (x-axis). The overall RMSD curves of WT and MUT proteins showed no significant differences, indicating that the mutation did not notably impact the overall stability of the BBS7 protein. A transient fluctuation observed around 40 ns in the WT protein was absent in the MUT protein, suggesting that the mutation might suppress local flexibility or conformational fluctuations.

C. Radius of gyration (Rg) curves for WT (blue) and MUT (red) BBS7 proteins during the 50 ns molecular dynamics simulations. The Rg values of both proteins remained consistently within the range of approximately 2.8 to 2.9 nm throughout the simulation, with no significant structural loosening or compaction observed. This indicates that the D252N mutation did not significantly affect the overall compactness and structural stability of the protein.

D. Solvent Accessible Surface Area (SASA) curves for WT and MUT BBS7 proteins throughout the 50 ns molecular dynamics simulations. Both proteins exhibited a slight decrease in SASA values during the initial phase (0–10 ns), followed by stabilization in subsequent phases. During the middle to later stages of the simulation, MUT proteins exhibited slightly higher SASA values compared to WT proteins, suggesting that the D252N mutation moderately increased protein surface exposure. However, this effect was not markedly pronounced.

**Figure S2: Differentiation of kidney lineage cells (KLCs) in 2D Culture**

A. Schematic of the KLCs differentiation protocol from hiPSCs, detailing treatment with 10 μM CHIR99021 (10 CHIR), 200 ng/mL FGF9 (200 FGF9), and 1 μg/mL heparin (1 Heparin).

B. Immunocytochemistry of nephron-like structures stained for markers of podocytes (PODXL), aquaporin 1 (AQP1) and collecting ducts (GATA3) on day 28; scale bar, 50 μm.

**Figure S3. BBS7 mutations impair KLC biological functions**

A. Schematic of the subculture protocol for differentiated KLCs.

B. Scratch assay demonstrating the impact of BBS7 mutations on KLC migration; cells from the same passage were used for both WT and MUT groups, with images captured at 0, 4, 8, and 12 hours post-scratch; dashed lines indicate scratch edges; scale bar, 200 μm.

C. Bar graph summarizing the percentage of wound closure at designated time points in the scratch assay; statistical significance is indicated as P < 0.05, P < 0.01.

D. Cell proliferation assay using the CCK-8.

**Figure S4. Structural analysis of interactions between BBS2, WT and MUT (D252N) BBS7 proteins, and the candidate E3 ubiquitin ligase MARCHF1.**

A-B. Docking models of the WT BBS7 protein (blue) (A) and mutant BBS7(D252N) protein (cyan) (B) interacting with the BBS2 protein (pink). Key residues involved in hydrogen bond interactions at the interfaces are highlighted in the enlarged views.

C-D. Structural models of the ternary complexes formed by wild-type BBS7 (purple) (A) or mutant BBS7(D252N) (cyan) (B), together with BBS2 (pink) and the candidate E3 ubiquitin ligase MARCHF1 (yellow). The wild-type BBS7 and MARCHF1 proteins show significant spatial overlap in their binding interfaces with BBS2. Upon introduction of the D252N mutation in BBS7, the interaction interface with BBS2 undergoes conformational rearrangement, exposing the MARCHF1 recognition site on BBS7. This structural change potentially enhances the ubiquitination and subsequent degradation of BBS7.

**Figure S5. Localization and distribution patterns of IFT88 in WT and MUT KLCs.**

A. Immunofluorescence staining showing localization of IFT88 in WT and MUT KLCs, co-stained with acetylated α-tubulin (cilia axoneme marker) and γ-tubulin (cilia basal body marker); scale bar, 10 μm.

B. Line graphs illustrating fluorescence intensity profiles of IFT88 along the axoneme of representative cilia from WT and MUT KLCs.

**Figure S6. Validation and analysis of primary cilia-related signaling pathway changes in WT and MUT KLCs.**

A. Validation of primary cilia PCR array results using RT-qPCR in KLCs (n = 3).

B. Immunofluorescence staining of GLI3 (white) in WT and MUT KLCs, co-stained with rootletin (red, ciliary base marker) and DAPI (blue, nuclear marker); scale bar, 5 μm.

C. Quantification of GLI3-positive cilia in WT and MUT KLCs. The percentage of GLI3-positive cilia was calculated as the ratio of GLI3-positive to acetylated α-tubulin-positive cilia (expressed as a percentage; n = 9).

D. Western blot analysis showing β-catenin protein expression levels in WT and MUT KLCs.

E. Immunofluorescence staining of β-catenin (white) with DAPI nuclear staining (blue) in WT and MUT KLCs; scale bar, 30 μm.

F. Quantification of β-catenin-positive nuclear foci per nucleus in WT and MUT KLCs. Statistical significance is indicated as **P* < 0.05, ***P* < 0.01, ****P* < 0.001; "ns" denotes not significant (n = 3).
